# Vitamin B6 Metabolism Determines T Cell Anti-Tumor Responses

**DOI:** 10.3389/fimmu.2022.837669

**Published:** 2022-02-17

**Authors:** David Bargiela, Pedro P. Cunha, Pedro Veliça, Iosifina P. Foskolou, Laura Barbieri, Helene Rundqvist, Randall S. Johnson

**Affiliations:** ^1^ Department of Physiology, Development and Neuroscience, University of Cambridge, Cambridge, United Kingdom; ^2^ Department of Cell and Molecular Biology, Karolinska Institute, Stockholm, Sweden

**Keywords:** vitamin B6, hypoxia, CD8+ lymphocytes, metabolism, immunotherapy

## Abstract

Targeting T cell metabolism is an established method of immunomodulation. Following activation, T cells engage distinct metabolic programs leading to the uptake and processing of nutrients that determine cell proliferation and differentiation. Redirection of T cell fate by modulation of these metabolic programs has been shown to boost or suppress immune responses *in vitro* and *in vivo*. Using publicly available T cell transcriptomic and proteomic datasets we identified vitamin B6-dependent transaminases as key metabolic enzymes driving T cell activation and differentiation. Inhibition of vitamin B6 metabolism using the pyridoxal 5’-phosphate (PLP) inhibitor, aminoxyacetic acid (AOA), suppresses CD8+ T cell proliferation and effector differentiation in a dose-dependent manner. We show that pyridoxal phosphate phosphatase (PDXP), a negative regulator of intracellular vitamin B6 levels, is under the control of the hypoxia-inducible transcription factor (HIF1), a central driver of T cell metabolism. Furthermore, by adoptive transfer of CD8 T cells into a C57BL/6 mouse melanoma model, we demonstrate the requirement for vitamin B6-dependent enzyme activity in mediating effective anti-tumor responses. Our findings show that vitamin B6 metabolism is required for CD8+ T cell proliferation and effector differentiation *in vitro* and *in vivo*. Targeting vitamin B6 metabolism may therefore serve as an immunodulatory strategy to improve anti-tumor immunotherapy.

## Introduction

Immunotherapy is an established treatment for cancer and targeting immune cell metabolism has emerged as a promising immunotherapeutic strategy (1, 2). Metabolic programs directly influence cell function and modulation of these programs has been shown to redirect T cell proliferation and differentiation. Metabolic control of T cell activity can thus be harnessed for therapeutic purposes by augmenting or suppressing immune responses *in vivo*. The identification of effective metabolic targets that confer cell-specific and durable immunomodulatory responses remains an ongoing priority.

In T cells, the hypoxia-inducible factor (HIF) transcription factors are master regulator of metabolic genes that direct cell fate and function (3–7). Metabolite availability, along with activation signals downstream of the T cell receptor (TCR) and co-stimulatory receptors, dynamically modulate HIF activity at the transcriptional, translational and post-translational level (8). In doing so, exogenous signals from the tumor microenvironment are able to direct HIF-driven transcriptional programs, leading to the promotion or suppression of effector T cell responses in a context-specific manner. Our group, and others, have shown that HIF-driven gene expression is required for effector CD8+ T cell differentiation but also maintenance of a memory T cell phenotype allowing for sustained anti-tumor immune responses (3, 4, 6, 9).

Here using an unbiased bioinformatic approach we identify vitamin B6-dependent enzymes as top metabolic hits in T cells following activation. We show that inhibition of vitamin B6 metabolism significantly limits T cell activation and differentiation *in vitro* and impairs anti-tumor immunity *in vivo*. Furthermore, we demonstrate that expression of PDXP, a key regulator of vitamin B6 levels, is HIF1-dependent in CD8^+^ T cells. Taken together these findings provide a mechanistic insight of vitamin B6 metabolism in T cell fate determination and support its use as a potential therapeutic target for anti-tumor immunotherapy.

## Materials and Methods

### Animals

Animal work was carried out under UK Home Office guidelines and the approval of the Regional Animal Ethics Committee of Northern Stockholm, Sweden. Mice were housed in a specific pathogen-free animal facility, provided with food and water *ad libitum*, and maintained on a 12-hour light-dark cycle at 21°C. Genotyping was performed with DNA from ear biopsies using commercial Transnetyx qPCR assays. C57Bl/6J mice were purchased from Janvier Labs. Donor TCR transgenic OT-1 mice (JAX 003831, The Jackson Laboratory) were crossed with mice bearing the CD45.1 congenic marker (JAX 002014, The Jackson Laboratory). All mice were backcrossed over ten generations to the C57BL/6J background. Male and female animals >6 weeks of age were used in experiments. Groups were assigned randomly for mouse experiments and the investigator was blinded to group assignment. No statistical methods were used to pre-determine sample size.

### Cell Lines

B16 F10 (mouse melanoma) cells were purchased from ATCC (CRL-6475). Ovalbumin-expressing B16 F10 (B16 F10 OVA) cells were generated in-house by co-transfection of the transposon vector pT2 encoding OVA, eGFP and neomycin phosphotransferase and the vector encoding transposase SB11. After three days, 400 μg/ml G418 (Gibco, 10131035) was added to culture media to select for transgene-expressing cells. Successful integration was confirmed by analyzing eGFP fluorescence by flow cytometry. Monoclonal OVA-expressing lines were derived by a process of limiting dilution. OVA presentation was confirmed by flow cytometry using a PE-labelled antibody against surface SIINFEKL bound to H-2Kb (clone 25- D1.16, BioLegend).

### Cell Culture

B16 F10 OVA cells were grown in T75 cell culture flasks (Sarstedt) in high-glucose DMEM supplemented with 10% fetal bovine serum (FBS), 1% penicillin/streptomycin and 0.75 mg/ml G418 (all Thermo Fisher). Mouse CD8+ T cells were grown in 48 well plates (Corning) in RPMI media supplemented with 10% fetal bovine serum (FBS), 1% penicillin/streptomycin, 55 μM 2-ME (all Thermo Fisher) and 100 units/ml recombinant human IL-2 (Roche). Compounds were pH corrected to pH 7.2-7.4 using sodium hydroxide or hydrochloric acid, as required, and added to wells at the time of initial seeding. Cells were cultured in humidified incubators with 5% carbon dioxide and 1% or 21% oxygen, as indicated in figure legends.

### Cell Count and Viability Measurement

Cells were either counted using an automated cell counter (BioRad TC20) with viability assessed by trypan blue exclusion and with LIVE/DEAD (Life Technologies) viability dye and absolute numbers were determined by flow cytometry using counting beads (Countbright, Life Technologies). Proliferation rate was calculated using the following formula: Proliferation Rate (Doublings per day) = log_2_ (final cell count/initial cell count)/number of days.

### Cell Division Analysis (CTV)

Cells were washed in PBS and incubated with 5 μM CellTrace Violet (Invitrogen C34557) in PBS for 30 minutes. RPMI media was then added for 5 minutes, after which the cells underwent centrifugation and were resuspended in fresh RPMI for onward culture. At the end of the experiment, samples were stained with Near-IR Dead Cell Stain Kit (ThermoFisher) and analyzed on a FACSCanto II flow cytometer (BD Biosciences). Cell division analysis was carried out by comparing cell division number and the proportion of cells in each division. Proliferation index is the total number of divisions divided by the number of cells that went into division and was calculated with FlowJo flow cytometry software.

### DNA Synthesis Analysis (EdU)

DNA synthesis was measured by quantifying incorporation of the nucleoside analog EdU (5- ethynyl-2 ´-deoxyuridine) in DNA using a click reaction coupled to a fluorophore. EdU was detected using the Click iT-EdU assay (Invitrogen C10634) according to the manufacturer’s instructions. In addition, samples were stained with Near-IR Dead Cell Stain Kit (ThermoFisher) and anti-mouse CD8 (clone: 53-6.7, BD Biosciences) and analysed on a FACSCanto II flow cytometer (BD Biosciences).

### CD8+ T Cell Isolation and Activation

CD8+ T cells were isolated from mouse spleens and purified using Microbeads conjugated to monoclonal anti-mouse CD8α (Ly-2; isotype: rat IgG2a) antibody (Miltenyi, 130-117-044), followed by magnetic bead isolation on a MACS column. CD8+ T cells were activated with anti-mouse CD3/CD28 Dynabeads (Thermo Fisher) at a 1:1 cell/bead ratio. After 3 days of culture, Dynabeads were removed using a magnet and cells were prepared for flow cytometry analysis or cultured for a further 4 days. OT-I CD8+ T cells from OT-I mouse spleens were activated with 1μg/ml SIINFEKL peptide for 48 hours. Following this, OT-1 T cells were washed twice with PBS and cultured for a further 5 days. All T cells were cultured in complete RPMI medium containing 2 mM glutamine, supplemented with 10% FBS, 1% Streptomycin, 55 μM β-mercaptoethanol (all Thermo Fisher) and 100 units/ml recombinant human IL-2 (Roche, 10 799 068 001).

### Vectors

DNA encoding a codon-optimized polycistronic peptide composed of mouse Thy-1.1 (AAR17087.1), picornavirus P2A (GSGATNFSLLKQAGDVEENPGP) and furin (RAKR) cleavage sequences and either wild-type/mutated mouse PDXP (NP_064667.2, wt; NP_064667.2:p.Ala194_Ala195delinsLysLys, mut) or wild-type/mutated mouse PDXK (NP_742146.1, wt; NP_742146.1:p.Ala228Thr, mut) was synthesized by Gene Art (Thermo Fisher). Cell surface peptides from Thy-1.1 were maintained to ensure proper subcellular localization. The coding sequences were cloned into the gamma retroviral vector pMP71, a gift from Christopher Baum (MHH, Hannover). Helper vector pCL-Eco (for ecotropic infection) was a gift from Inder Verma (Addgene; plasmid #12371). All protein sequences and accession numbers are available in **Supplementary Material**.

### Retroviral Transductions

Sub-confluent HEK293 cultures were transfected using FuGENE HD (Promega, #E2311/2) with 2.5 mg PDXP- or PDXK-encoding vectors and 1.5 mg helper vector pCL-Eco, for generation of mouse-tropic retroviral particles. Supernatant media containing retroviral particles were harvested 48 hours after transfection and used fresh. Retroviral supernatants were spun onto RetroNectin-coated wells (Takara) at 2,000 g for 2 hours at 32°C and replaced with activated mouse polyclonal CD8^+^ T cells in fresh RPMI supplemented with 100 U/mL IL2.

### *In Vivo* Tumor Chemotherapy Model

Female C57BL/6J mice, aged between 7 - 12 weeks, were injected subcutaneously with 500,000 B16 F10 OVA cells and tumor growth was monitored over time. After 10 days, mice received a daily intraperitoneal injection of PBS (control), 0.25 mM aminoxyacetic acid (AOA), 1 mM oxaloacetate (OAA) or 0.25 mM AOA + 1 mM OAA for 5 days and tumor growth was monitored thereafter until a humane endpoint was reached. Animals were assigned randomly to each experimental group. Tumors were measured blindly every 2-3 days (daily evaluations during injections) with digital calipers. Tumor volume was calculated using the formula: (*a* ^2^ x *b*) x 0.5 where *a* is the width and *b* is the length of the tumor.

### *In Vivo* Tumor Adoptive Immunotherapy Model

Splenocytes from female CD45.1+ OT-1 mice were activated with 1 μM SIINFEKL peptide and treated with vehicle (water), 0.25 mM aminoxyacetic acid (AOA, Sigma), 1 mM oxaloacetate (OAA, Sigma) or 0.25 mM AOA + 1 mM OAA in the presence of IL-2 (100 units/ml). After 48 hours, CD8+ OT-1 T cells were purified using magnetic negative selection beads (Miltenyi) and cultured for 5 further days with vehicle (water), 0.25 mM aminoxyacetic acid (AOA, Sigma), 1 mM oxaloacetate (OAA, Sigma) or 0.25 mM AOA + 1 mM OAA in the presence of IL-2 (100 units/ml). On day 7, one million OT-1 T cells were transferred intraperitoneally into tumor-bearing CD45.2+ C57BL/6J mice that had been subcutaneously injected with 500,000 B16 F10 OVA cells 7 days prior and then four days after that received an intraperitoneal injection of 300 mg/kg cyclophosphamide to achieve lymphodepletion. The expansion of OT-1 T cells in peripheral blood was evaluated from tail vein blood samples taken 7 days following OT-1 transfer. Tumor growth was evaluated until a humane endpoint was reached. Tumors were measured blindly every 2-3 days (daily evaluations during injections) with digital calipers and tumor volume was calculated using the formula: 0.5 x (*a*_2_ x *b*) where *a* is the width and *b* is the length of the tumor.

### Flow Cytometry

Single cell suspensions were stained with Near-IR Dead Cell Stain Kit (ThermoFisher) followed by surface, cytoplasmic and/or nuclear staining with the following fluorochorome- conjugated monoclonal antibodies: CD25 (PC61.5) purchased from ThermoFisher; CD44 (IM7), CD62L (MEL-14), CD8 (53-6.7), Fc Block (93), Granzyme B (QA16A02), ICOS (C398.4A), IFNγ (XMG1.2) and TNF⍺ (MP6-XT22) purchased from Biolegend. Staining of cytoplasmic and nuclear antigens was performed using the Fixation/Permeabilization kit (BD Biosciences) and the Transcription Factor buffer set (Invitrogen/eBiosciences), respectively. For staining of intracellular cytokines CD8+ T cells were re-stimulated with PMA, Ionomycin and Brefeldin A (all Sigma) for 4 hours. Samples were analysed on a FACSCanto II flow cytometer (BD Biosciences) and using FlowJo version 10 software (BD Biosciences). The gating strategy was as follows: FSC-A, SSC-A lymphocytes >> FSC-A, FSC-H singlets >> CD8 positive, LIVE/DEAD Negative.

### Metabolomics

Metabolomics experiments were previously performed using GC/MS and LC/MS/MS plat forms platforms (Metabolon Inc.) to determine intracellular metabolite concentrations. Raw values were normalized using cell counts or protein concentration as measured by the Bradford assay and rescaled to set the median to 1. Missing values were imputed using the minimum value. Principal component analysis (PCA) was carried out using the *prcomp* function, unsupervised hierarchical clustering was carried out using the *hclust* function, and Z scores and log fold changes were calculated in R version 3.6.1.

### RNAseq Analysis

CD8+ T cells were activated and cultured with vehicle (water) or 0.25 mM AOA in the presence of IL-2 for 7 days, then washed twice with ice cold PBS and lysed with RLT buffer containing 1% β-mercaptoethanol. Total RNA was subjected to quality control with Agilent Tapestation according to the manufacturer’s instructions. To construct libraries suitable for Illumina sequencing the Illumina TruSeq Stranded mRNA Sample preparation protocol which includes cDNA synthesis, ligation of adapters and amplification of indexed libraries was used. The yield and quality of the amplified libraries were analysed using Qubit by Thermo Fisher and the Agilent Tapestation. The indexed cDNA libraries were normalised and combined and the pools were sequenced on the Nextseq 550 for a 75-cycle v2.5 sequencing run generating 2x75 bp paired-end reads. Basecalling and demultiplexing was performed using CASAVA software with default settings generating Fastq files for further downstream mapping and analysis. Sequenced reads were mapped to the genome using the STAR aligner with default settings and uniquely mapped reads were counted. Normalised counts per million and differential gene expression were determined with DESeq2 (Love, 2014). Principal component analysis (PCA) was carried out using the R *prcomp* function, unsupervised hierarchical clustering was carried out using the R *hclust* function, and Z scores and log fold changes were calculated in R version 3.6.1. The raw RNAseq data are deposited in the GEO database under the series number GSE182999.

### Gene Set and Pathway Enrichment Analysis

Gene-set enrichment analysis (GSEA analysis) was performed on a complete ranked list of our genes using the fgsea R package and a previously described approach (Subramanian 2005). The following genesets from the Molecular Signature Database at the Broad Institute (https://www.gsea-msigdb.org/gsea/msigdb/index.jsp) were utilized: G2/M cell cycle checkpoint (HALLMARK_G2M_CHECKPOINT, ‘G2M checkpoint’), cell cycle related targets of E2F transcription factors (HALLMARK_E2F_TARGETS, ‘E2F targets’), genes involved in T cell activation (T_CELL_ACTIVATION, ‘T cell activation’), genes involved in T cell proliferation (GO_POSITIVE_REGULATION_OF_T_CELL_PROLIFERATION, ‘T cell proliferation’) and genes involved in T cell differentiation (GO_T_CELL_DIFFERENTIATION_INVOLVED_IN_IMMUNE_RESPONSE, ‘T cell differentiation’). Pathway enrichment analysis was performed using the ClusterProfiler R package and pathways from the Reactome database (https://reactome.org) in R version 3.6.1.

### Seahorse

Oxygen consumption rates (OCR) and extracellular acidification rates (ECAR) were measured on a Seahorse XFe96 Analyzer (Agilent) using 2 x10^5^ CD8+ T cells per well on a poly-D- lysine coated plate with XF RPMI media pH 7.4 (containing 10 mM glucose, 2 mM L- glutamine). Measurement were made under basal conditions and following addition of 0.25 mM AOA, 1 mM OAA, 1 μM oligomycin, 1.5 μM FCCP and 100 nm rotenone + 1 μM antimycin. All compounds were acquired from Sigma-Aldrich. For experiments involving *in Seahorse* activation of T cells, soluble anti-CD3 (5 μg/ml, clone:17A2) and anti-CD28 (5 μg/ml, clone:37.51) antibodies (both Biolegend) were injected from Port A. Relative ECAR and relative OCR measurements were calculated by dividing all measurements by the final baseline measurement (measurement immediately before the Port A injection).

### Immunoblotting

CD8+ T cells were collected, washed twice in ice cold PBS and lysed in UTB buffer with β- mercaptoethanol. Protein lysates were quantified with Bradford Assay reagent (BioRad) and separated by SDS-PAGE on Nu-Page 3-8% Tris acetate or 4-12% Bis-Tris gels (Life Technologies). Proteins were transferred to nitrocellulose membranes and blocked with Roti- Block (Carl Roth). Membranes were incubated with primary antibodies (FIH, 1:1000, Santa- Cruz, sc-271780; Tubulin, 1:10000, Abcam, ab6160) overnight at 4°C and then with infrared dye-conjugated secondary antibodies at room temperature. Imaging of membranes was carried out using an Odyssey imaging system (LICOR).

### T Cell Metabolic Gene and Protein Signature

We used microarray data from the Immunological Genome Project (ImmGen) (10) and proteomic data from the Immunological Proteomic Resource (ImmPRes) (11) to determine gene and protein expression in murine activated T cell populations at multiple timepoints. A false discovery rate (FDR) threshold of 5% was applied to determine differentially-expressed genes. To assemble a list of metabolic genes, we obtained all metabolic genes from the KEGG database (https://www.genome.jp/kegg/pathway.html) and supplemented this list with a manually curated list of genes encoding solute carrier (SLC) transporters (**Supplementary Data 1**). A T cell metabolic gene and protein signature was derived by overlapping gene and protein expression data with this metabolic gene list.

### HIF1 Metabolic Gene Signature

A list of HIF1-driven genes was generated using two previously published RNAseq datasets of *in vitro* culture CD4+ T cells with or without prolyl hydroxylase 1-3 (PHD WT and PHD KO, respectively, GSE85131) (3) and CD4+ T cells with or without HIF1 (HIF1 WT and HIF1 KO, respectively, GSE29765) (12). Prolyl hydroxylases (PHDs) regulate the abundance of HIF within cells by marking HIF molecules for downstream degradation in an oxygen-dependent manner. A FDR threshold of 5% was applied on differentially-expressed genes in both datasets (comparing WT vs KO) and genes that were both significantly up in the PHD KO T cells (vs PHD WT) and significantly down in the HIF1 KO T cells (vs HIF1 WT) were selected (**Supplementary Data 2**). To assemble a list of metabolic genes, we obtained all metabolic genes from the KEGG database (https://www.genome.jp/kegg/pathway.html) and supplemented this list with a manually curated list of genes encoding solute carrier (SLC) transporters (**Supplementary Data 1**). A HIF1 metabolic gene signature was derived from all genes present both in the HIF1-driven genes list and the metabolic gene lists (**Supplementary Data 3**).

### Statistical Analysis

Statistical analyses were performed in GraphPad Prism 8 software and R version 3.6.1. Pairwise comparisons of unpaired data were carried out using a two-tailed Student’s t-test with Welch’s correction, where appropriate. Pairwise comparison of paired data was carried out using a paired t-test. Multiple comparisons were carried out using one-way ANOVA with Dunnett’s multiple comparison test or using multiple t-tests with multiple comparison correction *via* the two-stage step-up method of Benjamini, Krieger and Yekutieli. Grouped data was assessed by two-way ANOVA, correcting with Tukey’s multiple comparisons test. Error bars shown represent s.e.m., unless otherwise stated in figure legends. Sample sizes were chosen based on previous experience of *in vitro* and *in vivo* experiments.

## Results

### Vitamin B6- (PLP-) Dependent Enzymes Are Highly Expressed in Activated T Cells

**Figure 1:**
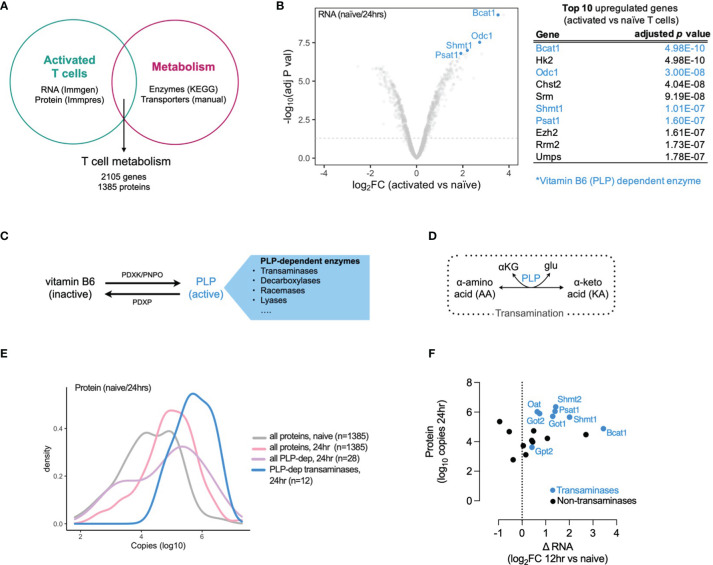
Vitamin B6 (PLP) dependent transaminases are highly expressed in activated T cells. **(A)** T cell metabolism gene and protein expression signature generated from overlap of publicly-available T cell activation mRNA (Immgen) and protein (Immpres) datasets with KEGG metabolic genes and manually-curated metabolic gene lists. **(B)** Top 10 upregulated genes in 24hr activated versus naïve CD8+ OT-1 T cells (Immgen), vitamin B6 (PLP) dependent enzymes highlighted in blue. **(C)** Vitamin B6 is converted to pyridoxal 5'-phosphate (PLP), its active form which is utilized as a cofactor by numerous PLP-dependent enzymes. **(D)** PLP-dependent transamination is required for interconversion between alpha-amino acids (AA) and alpha-keto acids (KA). **(E)** Distribution of protein copy number in naive and 24hr activated T cells (Immpres). **(F)** Gene and protein expression of PLP-dependent enzymes in activated T cells, PLP-dependent transaminases in blue; PLP-dependent non-transaminases in black.

To explore the role of metabolism in T cell activation and differentiation we generated a T cell metabolism gene and protein signature using publicly available datasets. We used microarray data from the Immunological Genome Project (ImmGen) (10) and proteomic data from the Immunological Proteomic Resource (ImmPRes) (11) to determine gene and protein expression in murine activated T cell populations at multiple timepoints. We overlapped this gene and protein expression signature with a list of metabolic enzymes (derived from all metabolic pathways in the Kyoto Encyclopedia of Genes and Genomes (KEGG) database) and solute carrier transporter (manual curation, **Supplementary Data 1**), to generate a T cell metabolism signature containing 2105 genes and 1385 proteins (**Figure 1A**) . Using this T cell metabolism signature, we determined which genes were most upregulated in T cells 24 hours post-activation when compared to naive cells. We found that 4 of the top 10 upregulated genes were vitamin B6 (pyridoxal 5’-phosphate, PLP) dependent enzymes, and the PLP-dependent transaminase, Bcat1, was the most significantly upregulated gene (adjusted p value = 4.98 x 10^-10^) (**Figure 1B**). Vitamin B6 is obtained from dietary sources and is converted to an active form, pyridoxal 5’-phosphate (PLP), by the action of pyridoxal kinase (PDXK) and pyridoxamine 5’-phosphate oxidases (PNPO). Pyridoxal phosphate phosphatase (PDXP) converts PLP back to its inactive form. PLP is utilized as a cofactor by numerous (>140) enzymes, including transaminases, which interconvert amino acids and ketoacids (**Figures 1C, D**). To further identify which PLP-dependent enzymes are present in T cells, we assessed gene and protein expression in T cells at timepoints following activation, dividing them as PLP-dependent transaminases and PLP-dependent non-transaminases (**Supplementary Figure 1A, B**). In addition, we assessed the gene and protein expression of PLP regulators (PDXK, PNPO and PDXP) in timepoints following activation (**Supplementary Figures 1A, B**). Overall, the expression of PLP-dependent transaminases was higher than PLP-dependent non-transaminases at both mRNA and protein levels and the increase in PLP-dependent transaminase expression occurred early following T cell activation (**Supplementary Figures 1A, B** and **Figures 1E, F**). At 12 hours following activation, there was an increase in gene expression of positive regulators of PLP levels (PDXP, PNPO) relative to naïve cells, whereas there was no change in gene expression of the negative regulator of PLP levels (PDXP). However by 24 hours after activation, gene and protein expression levels of both positive and negative regulators were similar to those found in naïve cells (**Supplementary Figures 1A, B**).

### Inhibition of PLP-Dependent Enzymes Limits T Cell Proliferation

**Figure 2:**
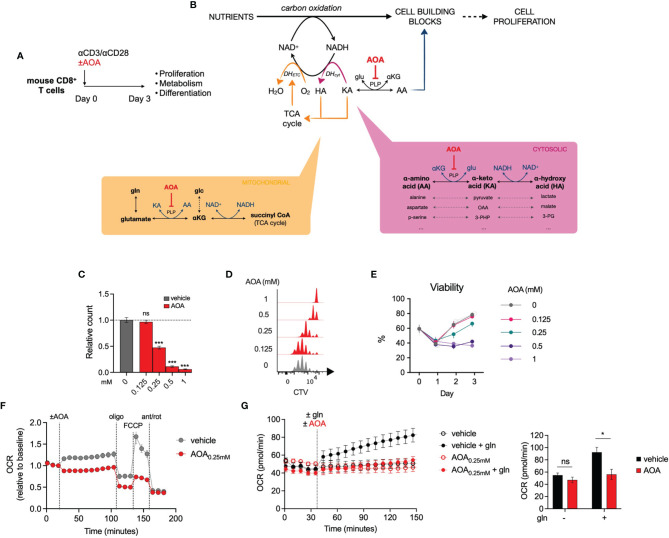
Inhibition of PLP-dependent enzymes with AOA limits cell proliferation **(A)** *In vitro* experimental design. Mouse CD8+ T cells were activated for 3 days and proliferation, metabolism and differentiation were profiled. **(B)** Schematic illustrating the contribution of PLP-dependent transaminases to cell proliferation and the site of action of the PLP inhibitor, aminoxyacetic acid (AOA). **(C, D)** Relative cell count **(C)** and cell division **(D)** of CD8+ T cells following 48 hours of treatment with AOA (n = 8 mice), **(E)** Viability of CD8+ T cells during 3 days of activation with different concentrations of AOA (n = 4 mice) **(F)** Seahorse metabolic profiling showing oxygen consumption rate of CD8+ T cells after addition of vehicle or 0.25mM AOA (n = 8 mice). **(G)** Oxygen consumption rate of glutamine- (gln-) depleted CD8+ T cells following treatment with the amimo acid, glutamine, and/or AOA (n = 3). Data representative of at least two independent experiments. Error bars denote s.e.m. One-way ANOVA with Dunnett's multiple comparisons test **(C)**. Two-way ANOVA with Tukey's multiple comparisons test **(G)**. *p < 0.05, ***p < 0.001; ns, non significant; gln, glutamine; MFI, mean fluorescence intensity.

To explore the role of PLP-dependent enzymes in T cell function, we treated activated mouse CD8+ T cells with aminoxyacetic acid (AOA), a well-characterized PLP inhibitor (**Figure 2A**). Given that PLP-dependent transaminases are involved in NAD regeneration and nucleotide synthesis (**Figure 2B**) and have previously been demonstrated to be important in cell proliferation and differentiation (13–15), we predicted that AOA treatment would restrict *in vitro* CD8+ T cell expansion. We measured the proliferation of CD8+ T cells following 3 days of AOA treatment and found that AOA-induced PLP inhibition resulted in a dose-dependent decrease in both cell counts (**Figure 2C**) and cell division (**Figure 2D**). T cell viability was assessed on each day of culture and AOA concentrations greater than 0.25mM were shown to markedly reduced T cell viability (**Figure 2E**). To assess the contribution of PLP enzymes to NAD/NADH homeostasis within mitochondria, we measured oxygen consumption rate (OCR) in activated T cells and found that AOA treatment limited both basal and maximal OCR (**Figure 2F**). To confirm that this was secondary to PLP-dependent transaminase activity we combined treatment of AOA with glutamine, which contributes carbons to the TCA cycle *via* glutamate dehydrogenase and/or PLP-dependent glutamate-oxaloacetate transaminase activity. In vehicle-treated CD8+ T cells that were initially in glutamine-free media, the addition of glutamine led to a significant increase in OCR, whereas addition of glutamine to AOA- treated activated CD8+ T cells resulted in a limited increase in OCR, comparable to cells remaining in glutamine-free media (**Figure 2G**). These results suggest that PLP-dependent enzyme activity is required for T cell proliferation and to support mitochondrial oxidation through the action of transaminases.

### Inhibition of PLP-Dependent Enzymes Restricts Effector T Cell Differentiation and Results in Metabolic Reprogramming

**Figure 3:**
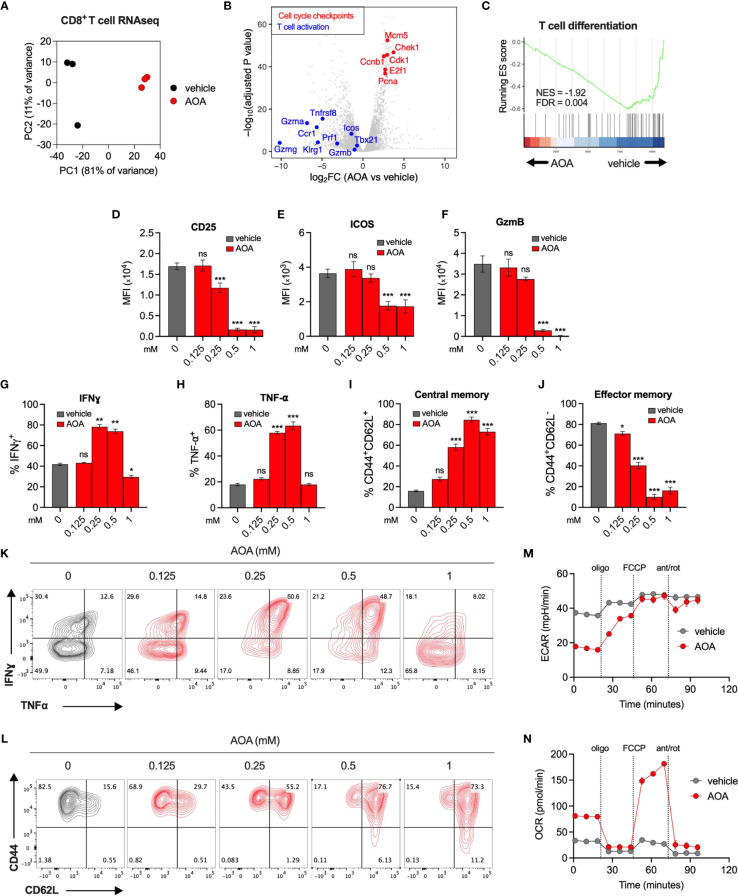
Inhibition of PLP-dependent enzymes limits T cell activation and differentiation. **(A)** PCA of RNAseq expression data from CD8+ T cells treated with vehicle or AOA. **(B)** Volcano plots of selected genes that significantly increased (red, adj *p* < 0.05) or significantly decreased (blue, adj *p*<0.05) following AOA treatment. **(C)** Gene set enrichment analysis (GSEA) plots showing enrichment of T cell differentiation genes in vehicle- versus AOA-treated samples **(D–H, K)** Phenotypic characterization of activation markers **(D–F)** and effector cytokines **(G, H, K)** in CD8+ T cells following 3 days of AOA treatment using flow cytometry (n = 4-8 mice). **(I, J, L)** CD44 and CD62L surface expression on CD8+ T cells following 7 days of AOA treatment (n = 8 mice). **(M, N)**. Seahorse metabolic profiling showing extracellular acidification rate **(M)** and oxygen consumption rate **(N)** of CD8+ T cells after activation and treatment with vehicle or 0.25mM AOA for 7 days (n = 8 mice). Data are representative of at least two independent experiments. Error bars denote sem One way ANOVA with Dunnett's multiple comparisons test **(D–J, M, N)** *p < 0.05, ***p < 0.001; ns, non significant; MFI, mean fluorescence intensity.

To understand the effect of PLP inhibition on immune cell transcriptional programs, we carried out RNA sequencing of vehicle- or AOA-treated (0.25mM) CD8+ T cells. AOA treatment resulted in a distinct gene expression profile (**Figure 3A** and **Supplementary Figure 2A**), with significantly reduced expression of genes associated with T cell activation and effector function, and significantly increased expression of genes associated with cell cycle checkpoints (**Figures 3B, C**). We confirmed these findings using pathway analysis assessing the top 10 most enriched Reactome pathways in AOA-treated cells, which all related to cell cycle processes (**Supplementary Figure 2B**). Furthermore, using gene set enrichment analysis (GSEA) we found selective enrichment of genes associated with the G2/M cell cycle checkpoint in AOA-treated cells and enrichment of T cell activation and proliferation in vehicle-treated T cells (**Supplementary Figure 2D, E**). Next, to correlate these gene expression changes to changes in protein levels, we profiled key cell fate markers using flow cytometry. Treatment of CD8+ T cells with AOA resulted in a dose-dependent reduction of the activation markers CD25, ICOS and GzmB (**Figures 3D**–**F**), while intermediate doses of AOA boosted levels of the effector cytokines, Interferon gamma (IFNγ) and Tumor necrosis factor alpha (TNF-⍺) (**Figures 3G, H, K**). AOA treatment also resulted in a dose-dependent suppression of cell differentiation assessed by CD44/CD62L expression (**Figures 3I, J, L**). AOA treatment increased the central memory (CD44+CD62L+) population (**Figure 3I**) and reduced the effector memory (CD44+CD62L-) population (**Figure 3J**). To assess the metabolic consequences of chronic PLP inhibition we treated CD8+ T cells with vehicle or AOA for 7 days and performed metabolic profiling using Seahorse. Chronic AOA treatment suppressed basal, but not maximal, extracellular acidification (**Figure 3M**) while boosting basal and maximal mitochondrial oxidative activity (**Figure 3N**). Overall, this indicated that PLP inhibition with AOA restricts T cell differentiation programs and results in metabolic reprogramming following chronic treatment.

### PLP Inhibition Limits *In Vivo* Anti-Tumor T Cell Responses

**Figure 4:**
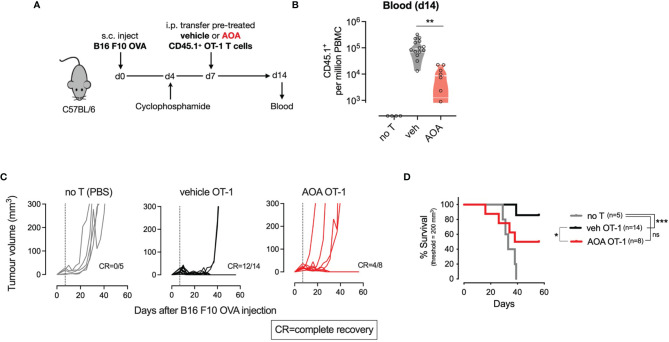
PLP-dependent enzymes are essential for *in vivo* T cell proliferation and anti-tumour responses. **(A)** Schematic outlining *in vivo* adoptive transfer experiment - C57B1/6 mice bearing B16 F10 OVA tumours were lymphodepleted and injected on day 7 with PBS (no T) or CD45.1 OT-1 T cells pre-treated with vehicle or AOA. **(B)** *In vivo* expansion of transferred CD45.I+ OT-1 cells measured from tail vein blood sample on day 14 (7 days post-transfer). **(C)** Tumor growth curves in mice receiving intraperitioneal injection of PBS (no T), vehicle or AOA-treated CD45.1- OT-1 cells at day 7 following B16 F1O OVA injection (dotted line). **(D)** Kaplan-Meier plot showing survival time to tumor volume 200 cubic millimeters. Shaded areas indicate s.e.m. One-way ANOVA with Dunnett's multiple comparisons test **(B)**. Logrank test **(C)**. *p < 0.05, **p < 0.01, ***p < 0.001; ns, non significant; i.p./I.P, intraperitoneal; PBS, phosphate-buffered saline; s.c., subcutaneous.

To assess whether AOA-induced inhibition of PLP-dependent enzymes could be leveraged *in vivo* as systemic chemotherapy by inhibiting tumor cell proliferation, we measured the growth of tumors in mice treated with AOA. C57BL/6 mice were injected subcutaneously with B16 F10 melanoma cells expressing ovalbumin (B16 F10 OVA) and tumor growth was monitored over time. After 10 days, a daily intraperitoneal injection of AOA alone was delivered for 5 consecutive days, and monitoring of tumor growth was continued thereafter (**Supplementary Figure 3A**). No significant difference in tumor growth was noted in vehicle- and AOA-treated mice over time (**Supplementary Figures 3B, C**). Next, to more specifically delineate the role of T cell PLP-dependent enzymes in anti-tumor responses, we used an adoptive transfer immunotherapy model. We treated CD45.1^+^ OT-I T cells for 7 days *in vitro* with vehicle or AOA then transferred these cells into mice that had previously received a subcutaneous injection of B16 F10 OVA cells followed by lymphodepletion with cyclophosphamide (**Figure 4A**). We measured the expansion of CD45.1^+^ OT-I T cells in the blood 7 days after T cell transfer and found that AOA-treated T cells expanded significantly less *in vivo* compared to vehicle-treated T cells (**Figure 4B**). Subsequent control of tumor growth was significantly improved in mice that received vehicle-treated OT-1 T cells where 12/14 (86%) mice achieved complete recovery, whereas only 4/8 (50%) mice receiving AOA-treated OT-1 T cells achieved complete recovery (**Figure 4C**). Survival of mice receiving vehicle-treated OT-1 T cells was significantly improved compared to mice receiving PBS alone (no T cells), whereas mice receiving AOA-treated T cells had no significant survival advantage over mice receiving PBS alone (**Figure 4D**). These results indicate that inhibition of PLP-dependent enzymes with AOA restricts *in vivo* T cell proliferation and anti-tumor function.

### Hypoxia and HIF1 Transcriptional Activity Modulate PLP Metabolism

**Figure 5:**
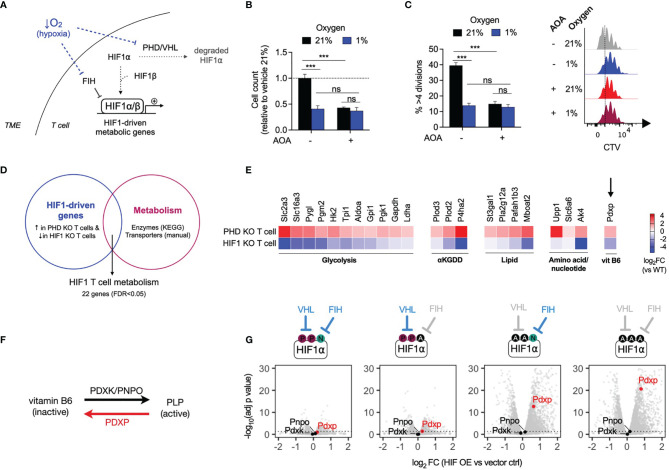
HIF1 drives expression of pyridoxal phosphate phosphatase (Pdxp) **(A)** Schematic of low oxygen levels (hypoxia) within the tumour microenvironment inhibiting the regulatory activity of oxygen-dependent HIF hydroxylases within T cells, leading to increased expression of HIF1-driven metabolic genes. **(B, C)** Relative cell count **(B)** and cell division **(C)** of CD8+ T cells after 3 days of treatment with AOA in 21% or 1% oxygen (n = 4 mice). **(D)** HIF1 T cell metabolism gene expression signature generated from overlap of publicly-available microarray datasets of PHD knockout (PHD KO, GSE85131) and HIF1 knockout (HIF1 KO. GSE29765) T cells with KEGG metabolic genes and manually curated metabolic gene lists. **(E)** Gene expression (log2 fold change versus wild-type) of 27 HIF1 metabolic signature genes that are significantly upregulated in PHD KO T cells and significantly downregulated in HIF1 KO T cells (FDR < 0.05). **(F)** Schematic of conversion between inactive vitamin B6 derivates and active pyridoxal 5-phosphate (PLP) form, by pyridoxal kinase (PDXK), pyridoxal oxidase (PNPO) and pyridoncal phosphate phosphatase (PDXP). **(G)** Volcano plots of *Pdxp*, *Pnpo* and *Pdxk* expression following topic expression of HIF1 in CDS-T cells-Four expression constructs with differing regulation were compared from left to nght, VHL and FIH regulated only VHL regulated, only FIH regulated and no VHL or FH regulation (gray indicates no regulation) Error bears denote s.e.m. Two-way ANOVA with Tukey’s multiple comparisons test **(B, C)** ***P < 0.001; ns, non significant.

As tumors grow, their consumption of oxygen can outstrip its local supply, resulting in limited oxygen availability for infiltrating immune cells. Low oxygen levels (hypoxia) restrict anti-tumor T cell proliferation, in part through inhibition of oxygen-dependent HIF hydroxylases resulting in upregulation of HIF1-driven metabolic gene expression (16) (**Figure 5A**). To assess the effects of hypoxia on T cell proliferation, mouse CD8+ T cells were treated with AOA and cultured in 21% and 1% oxygen. T cells cultured in 1% oxygen (hypoxia) grew significantly less compared to T cells culture in 21% (normoxia). However, in contrast to cells grown in 21% oxygen, addition of AOA to cells cultured at 1% oxygen resulted in no additional growth disadvantage (**Figures 5B, C**), suggesting a potentially common mechanism underlying growth suppression mediated by hypoxia and PLP inhibition. To determine whether HIF1-driven metabolic gene expression may be a common factor linking growth suppression caused by hypoxia and PLP inhibition, we generated a T cell HIF metabolism gene signature from publicly available datasets. Using microarray and RNAseq datasets we selected HIF1-driven genes, defined as genes that were both significantly increased in prolyl hydroxylase 1-3 (PHD) knockout T cells (PHD KO, GSE85131) in which HIF1 is constitutively active and significantly decreased in HIF1 knockout T cells (HIF1 KO, GSE29765) (FDR<0.05 for both, **Supplementary Data 2**). We overlapped this gene and protein expression signature with a list of KEGG metabolic enzymes (and solute carrier transporter (by manual curation, **Supplementary Data 1**), to generate a HIF1 T cell metabolism signature containing 22 genes (**Figure 5D** and **Supplementary Data 3**). In agreement with previous reports of HIF1 metabolic targets, the signature included genes encoding glucose transporters, such as *Slc2a3*; glycolysis genes, such as *Hk2* and *Ldha*, and a gene encoding the lactate transporter, *Slc16a3* (**Figure 5E**). In addition, the gene encoding pyridoxal phosphate phosphatase (*Pdxp*), was found to be both significantly increased in PHD-deficient cells and significantly decreased in HIF1-deficient T cells. PDXP regulates vitamin B6-dependent enzymes by removing a phosphate group from pyridoxal-5-phosphate (PLP), the active form of vitamin B6, and thus inactivates it (**Figure 5F**). To confirm which of the two major HIF isoforms, HIF1 or HIF2, is responsible for driving expression of *Pdxp*, we assessed the expression of the PLP regulator genes *Pdxp*, *Pdxk* and *Pnpo* in mouse CD8+ T cells that ectopically expressed varying levels of HIF1 or HIF2 (GSE166758) (7). Ectopic expression of HIF1, but not HIF2, significantly increased the expression of *Pdxp* in a dose-dependent manner, while *Pdxk* and *Pnpo* were not significantly affected by either HIF isoform (**Figure 5G** and **Supplementary Figure 4A**). Finally, we assessed the effect of directly modulating the endogenous PLP regulators, PDXP and PDXK, in normoxia and hypoxia. Ectopic overexpression of catalytically active *Pdxk* in CD8+ T cells augmented proliferation in 21% oxygen. Whereas, overexpression of catalytically inactive *Pdxk (mutPdxk)*, *Pdxp* or catalytically inactive *Pdxp (mutPdxp)* had no significant effect on T cell proliferation at 21% oxygen (**Supplementary Figure 4E**). In contrast, overexpression of *Pdxp*, but not *mutPdxp*, *Pdxk* or *mutPdxk*, significantly increased T cell proliferation in 1% oxygen (**Supplementary Figure 4F**). These results show that the effects of PLP inhibition on T cell proliferation are oxygen-dependent and that HIF1, but not HIF2, activity drives the expression of *Pdxp*, an endogenous PLP inhibitor.

### HIF1 Suppresses PLP-Dependent Enzymes Activity

**Figure 6:**
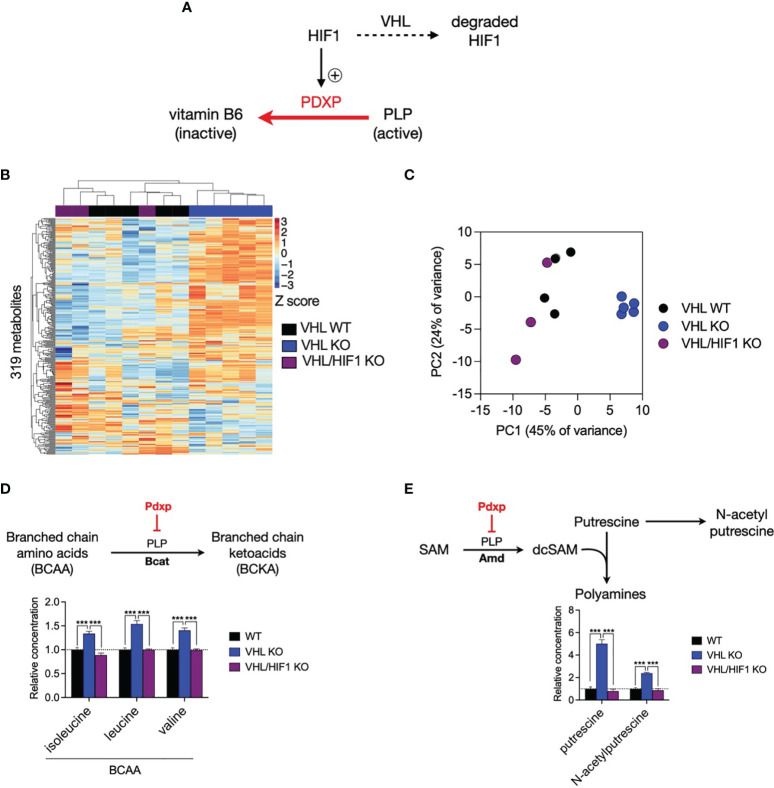
The VHL/HIF1 axis modulates the activity of PLP-dependent enzymes. **(A)** Schematic of VHL- and HIF1-dependent regulation of PDXP and PLP-dependent enzymes. **(B)** Heatmap of metabolite concentrations in double-floxed VHL (VHL WT, n = 5), VHL-/- (VHL KO, n-4) and VHL-/- HIF-/- (VHL/HIF1 KO, n = 3) CD8+ T cells. **(C)** Principal component analysis (PCA) of VHL WT, VHL KO and VHL/HIF1 KO CD8+ T cell samples. **(D)** Schematic of PLP-dependent branched chain amino acid (BCAA) metabolism and bar graph indicating relative concentration of BCAAs in VHL WT, VHL KO and VHL HIF1 KO CD8+ T cells. **(E)** Schematic of PLP-dependent polyamine biosynthesis and bar graph indicating relative concentration of putrescine and N-acetylputrescine in VHL WT, VHL KO and VHL/HIF1 KO CD8+ T cells. Error bars denote s.e.m. One-way ANOVA with Dunnett's multiple comparisons test **(D, E)**. ***p < 0.001; ns, non- significant; SAM, S-adenosylmethionine; desAM, decarboxylate S-adenosylmethionine; VHL, von Hippel-Lindau.

To address whether HIF1-driven *Pdxp* expression alters the activity of PLP-dependent enzymes in T cells, we performed metabolomic profiling using cell-specific knockouts of HIF1 and von Hippel Lindau (VHL, a negative regulator of HIF abundance) to reciprocally modulate HIF1 levels in CD8+ T cells (**Figure 6A**). We measured the intracellular concentrations of 319 metabolites and noted that the majority of variation in metabolite levels between VHL wild-type (VHL WT) and VHL knockout (VHL KO) cells was reversed upon additional deletion of HIF1 (VHL/HIF1 KO), implicating HIF1 as the major driver of metabolic reprogramming in VHL KO CD8+ T cells (**Figures 6B, C**). PLP-dependent enzymes activity was assessed by selecting rate-limiting, irreversible PLP-catalysed reactions, for which an increase in PDXP would reduce PLP activity and thus the reaction rate, resulting in substrate accumulation. As predicted, the substrates of these reactions were significantly increased in VHL KO cells where HIF1 is elevated and significantly decreased to WT levels upon deletion of HIF1 in VHL/HIF1 KO cells (**Figures 6D, E**). These findings suggest that HIF1-driven PDXP results in inhibition of PLP-dependent enzymes.

## Discussion

In this work we have shown that vitamin B6 (PLP-) dependent enzymes are significantly upregulated in activated T cells and influence T cell fate. Targeting of PLP enzymes, with the inhibitor AOA, restricts proliferation and differentiation of T cells both *in vitro* and *in vivo*, and results in impaired anti-tumor immune responses in mice. We also show that the vitamin B6 phosphatase, PDXP, downregulates PLP-dependent enzymes in an oxygen- and HIF1-dependent manner in T cells, providing a mechanistic link between hypoxia signaling and T cell adaptation within the tumor microenvironment.

PLP enzyme activity was assessed by treating cells with AOA, a well-characterized PLP inhibitor. AOA treatment profoundly limits T cell proliferation and differentiation, and these changes were ascribed to suppression of PLP-dependent transaminases, based on post-activation gene and protein expression profiles and metabolic characterization of CD8+ T cells. However, it is possible that inhibition of other non-transaminase PLP-dependent enzymes expressed in T cells may also contribute to this phenotype, as their activity would also be affected by AOA treatment. Insights on the specificity of AOA-induced PLP inhibition can be gleaned from previous work by Xu and colleagues using mouse CD4+ T cells (14). In their study, AOA was selected from a small molecule screen of inhibitors of effector CD4+ differentiation and subsequently confirmed to limit effector Th17 responses both *in vitro* and *in vivo*. Interestingly, they were able to replicate their AOA-induced phenotype by genetically-targeting a single PLP-dependent transaminase, GOT1, suggesting a central role for this enzyme in supporting proliferation and differentiation in T cells (14). GOT1 is highly expressed in both CD4+ and CD8+ T cells (10, 11) and forms part of the malate-aspartate shuttle, a crucial regulator of NAD/NADH, αKG and aspartate levels between the mitochondria and cytosol, which collectively influence T cell proliferation and fate (17).

When transferred *in vivo*, AOA-treated T cells proliferate less and offer poorer tumor growth control when compared to vehicle-treated T cells. A similar effect was noted by Xu and colleagues using a mouse model of experimental autoimmune encephalitis (EAE), in which AOA treatment suppressed effector CD4+ T cell differentiation leading to a reduction in inflammation-driven disability (14). The blunted effector response noted in the adoptive transfer tumor model may therefore be a carry-over effect from prior *in vitro* AOA treatment – although transferred T cells are no longer exposed to AOA *in vivo*, the inhibition of the PLP system may not be entirely reversed, resulting in continuing suppression of proliferation and differentiation. A previous study, using a mouse model of tumor immunotherapy, found that *in vivo* transfer of T cells that were previously treated with AOA *in vitro* were more effective at eliminating tumors when transferred *in vivo* (18). Importantly, however, the authors cultured cells in media that was supplemented with the ketoacid pyruvate, which may have eliminated the effects of AOA inhibition through direct interaction between the ketoacid and AOA, resulting in the formation of a stable ketoxime, as has been previously described (19–22). In addition, T cells were kept in RPMI media without AOA for 24 hours prior to transfer *in vivo*, which given the presence of supplementary vitamin B6 in RPMI media, may have also contributed to the reversal of previous AOA-induced inhibition. Notably, following injection of AOA directly into tumor-bearing mice we found no significant difference in tumor control when compared to vehicle-injected mice. This may be due to combined effect, with suppression of proliferation in both tumor cells and anti-tumor effector T cell populations, resulting in a minimal overall difference in tumor growth patterns. In addition, given that the *in vitro* effects of modulating PLP levels by overexpressing PLP regulators are oxygen-dependent, it is possible that PLP inhibition *in vivo* may have differing effect on T cell proliferation depending on whether the cells are exposed to higher oxygen tensions in circulation versus within hypoxic tumors.

In contrast to cells cultured in 21% oxygen, PLP inhibition does not significantly restrict proliferation of primary or transformed T cell populations cultured in 1% oxygen. In fact, overexpression of PDXP, the endogenous negative regulator of PLP, within T cells boosts their proliferation in 1% oxygen. In keeping with this observation, a previous CRISPR screen in Jurkat cells assessing metabolic genes required to sustain proliferation following partial electron transport chain (ETC) inhibition (13), identified the PLP-dependent transaminase GOT1, and the PLP-boosting enzyme PDXK, as top hits (13). However, a later study utilizing a CRISPR library targeting all genes in combination with complete ETC inhibition, no longer demonstrated a benefit of boosting PLP activity, and instead found loss of VHL and increased HIF levels as the most significant alteration supporting proliferation (23). Our results, paired with these findings, suggest a model in which proliferation is supported by ETC activity insofar as demands for reducing equivalents (e.g. NAD^+^ for glycolysis) and anaplerotic substrates (e.g. aspartate for nucleotide synthesis) can be met by boosting oxygen- and PLP-dependent processes, such as the malate-aspartate shuttle and one-carbon metabolism. When proliferative demands exceed ETC capacity, as in the case of hypoxia, HIF-driven suppression of PLP-dependent enzymes by increasing PDXP, alongside upregulation of glycolytic enzymes to support cytosolic NAD+ regeneration and ATP production, may serve as an adaptive mechanism to support continued T cell survival and proliferation under low oxygen tensions.

This is the first report, to our knowledge, identifying PDXP as a HIF1-driven gene in T cells, although previous studies have provided suggestive evidence in other cell types. *Pdxp* gene expression is increased in murine hepatic stellate cells cultured in hypoxia (24), in VHL-deficient murine endothelial fibroblasts (25) and in neuroendocrine tumors lacking *Siah2*, a ubiquitin ligase that modulates HIF stability (26). Furthermore, a chromatin immunoprecipitation (ChIP) assay in U98 glioma cells demonstrated HIF1-binding at the *Pdxp* gene promoter (27), though a further HIF1 ChIP study profiling other cancer cell types failed to detect binding, suggesting that HIF activity at this locus may be cell-type specific (28). In addition, HIF1-driven suppression of PLP enzymes *via* PDXP upregulation likely depends on the timing and duration of HIF1 activity. HIF-1 is stabilized *transiently* in T cells within the first 24 hours following activation (6). Over this time period there is minimal change in expression of the negative regulator PDXP. Instead, *prolonged* HIF1 stabilization, as would occur following HIF1 overexpression or during exposure to chronic hypoxia within the tumor microenvironment, may be required for robust upregulation of PDXP in T cells. This is further supported by our metabolomics analysis of VHL KO CD8+ T cells that have prolonged HIF activity and thus also expected to have robust Pdxp activity. In keeping with this, we see evidence of PLP inhibition in VHL KO T cells, with substrate accumulation occurring in irreversible, PLP-dependent metabolic reactions that is entirely reversed upon additional loss of HIF1.

In contrast to AOA treatment, indirect modulation of PLP levels by overexpression of the negative regulator of PLP levels, *Pdxp*, did not significantly alter T cell proliferation in 21% oxygen. Whereas overexpression of the positive regulator of PLP levels, *Pdxk*, significantly increased proliferation in 21% oxygen. This discrepancy may be a consequence of the timing of vector overexpression – to maximize transduction efficiency, T cells were transduced 24 hours post-activation - rather than a lack of functional activity. As initially demonstrated, the expression of PLP-dependent enzymes and the positive regulator, PDXK, but not the negative regulator PDXP, occurs early following activation and this expression profile may be both necessary and sufficient to support later proliferation. In line with this, previous studies using whole body knockout of *Pdxp* in mice, have achieved a 3-fold elevation of PLP levels in a variety of tissues, including brain, skeletal muscle and red blood cells (29). To extend our investigation of the role of vitamin B6 kinase/phosphatase regulators in immune cell activity, we intend to generate T cell-specific double-floxed PDXP and PDXK dLck Cre knockout mouse models. T cells from these mice will have fixed defects in vitamin B6 metabolism regulators, allowing characterization of the role of these regulators in determining PLP-dependent enzyme activity during early T cell activation. These mice will be further crossed with HIF1 double-floxed mice to study PLP activity in the context of varying oxygen and HIF1 levels. These models will assist in further exploration of possible interactions between PLP and HIF metabolism, such as the effect of PLP-dependent transaminase activity on aKG levels and subsequent downstream modulation of HIF activity *via* aKG-dependent HIF hydroxylases.

Limiting PLP levels through dietary deficiency of vitamin B6, leads to impairment of T cell responses in mice and humans (30–32), whereas vitamin B6 supplementation is effective in boosting cell mediated immune responses (33), particularly in elderly patients (32, 34). Although there are few clinical trials available to assess causality of vitamin B6 levels and cancer outcomes, epidemiological studies have detected an inverse relationship between plasma PLP levels and risk of colorectal and lung cancer (35–37). Future work will evaluate the therapeutic effect of dietary vitamin B6 depletion and supplementation in tumor-bearing mice. This will provide further insight into the role of PLP enzymes as modulators of cell proliferation and differentiation, and the potential of PLP modulation as a T cell immunotherapy strategy.

## Data Availability Statement

The datasets presented in this study can be found in online repositories. The names of the repository/repositories and accession number(s) can be found in the article/**Supplementary Material**.

## Ethics Statement

The animal study was reviewed and approved by Regional Animal Ethics Committee of Northern Stockholm, Sweden.

## Author Contributions

DB wrote and edited the manuscript, designed and carried out experiments, interpreted experiments and organized results. HR, PV, PC, IF, and LB wrote the manuscript and carried out experiments. RJ supervised and wrote the manuscript. All authors contributed to the article and approved the submitted version.

## Funding

Funding has been provided by the Wellcome Trust (211143/Z/18/Z), Swedish Cancer Society (Cancerfonden), the Swedish Childhood Cancer Fund (Barncancerfonden), and the Swedish Research Council (Vetenskapsrådet).

## Conflict of Interest

The authors declare that the research was conducted in the absence of any commercial or financial relationships that could be construed as a potential conflict of interest.

## Publisher’s Note

All claims expressed in this article are solely those of the authors and do not necessarily represent those of their affiliated organizations, or those of the publisher, the editors and the reviewers. Any product that may be evaluated in this article, or claim that may be made by its manufacturer, is not guaranteed or endorsed by the publisher.

## Supplementary Material

Supplementary Data Sheet 1 Solute carrier (Slc) transporter genes (manual curation).

Supplementary Data Sheet 2 HIF1-driven genes in T cells – genes sig ↑ in PHD KO vs PHD WT T cells AND sig ↓ in HIF1 KO vs HIF WT T cells (adjusted *p*<0.05).

Supplementary Data Sheet 3 HIF1 metabolic gene signature (adjusted *p*<0.05).

Supplementary Data Sheet 4 Peptides sequences for vectors.
