# Supplementary material for: Vitamin B6 Metabolism Determines T Cell Anti-Tumor Responses
Source: Front Immunol. 2022 Feb 17;13:837669. doi: 10.3389/fimmu.2022.837669 (PMC8891565; doi:10.3389/fimmu.2022.837669)
Supplement: Supplementary Data Sheet 1 — Solute carrier (Slc) transporter genes (manual curation). [file DataSheet_1.pdf]

__Supplementary Data 1__ **Solute carrier (Slc) transporter genes (manual curation)**

Slc10a1	Slc16a5	Slc22a2	Slc25a22	Slc26a6
Slc10a2	Slc16a6	Slc22a20	Slc25a23	Slc26a7
Slc10a3	Slc16a7	Slc22a21	Slc25a24	Slc26a8
Slc10a4	Slc16a8	Slc22a22	Slc25a25	Slc26a9
Slc10a5	Slc16a9	Slc22a23	Slc25a26	Slc27a1
Slc10a6	Slc17a1	Slc22a26	Slc25a27	Slc27a2
Slc10a7	Slc17a2	Slc22a27	Slc25a28	Slc27a3
Slc11a1	Slc17a3	Slc22a28	Slc25a29	Slc27a4
Slc11a2	Slc17a4	Slc22a29	Slc25a3	Slc27a5
Slc12a1	Slc17a5	Slc22a3	Slc25a30	Slc27a6
Slc12a2	Slc17a6	Slc22a30	Slc25a31	Slc28a1
Slc12a3	Slc17a7	Slc22a4	Slc25a32	Slc28a2
Slc12a4	Slc17a8	Slc22a5	Slc25a33	Slc28a3
Slc12a5	Slc17a9	Slc22a6	Slc25a34	Slc29a1
Slc12a6	Slc18a1	Slc22a7	Slc25a35	Slc29a2
Slc12a7	Slc18a2	Slc22a8	Slc25a36	Slc29a3
Slc12a8	Slc18a3	Slc23a1	Slc25a37	Slc29a4
Slc12a9	Slc18b1	Slc23a2	Slc25a38	Slc2a1
Slc13a1	Slc19a1	Slc23a3	Slc25a39	Slc2a10
Slc13a2	Slc19a2	Slc23a4	Slc25a4	Slc2a12
Slc13a2os	Slc19a3	Slc24a1	Slc25a40	Slc2a13
Slc13a3	Slc1a1	Slc24a2	Slc25a41	Slc2a2
Slc13a4	Slc1a2	Slc24a3	Slc25a42	Slc2a3
Slc13a5	Slc1a3	Slc24a4	Slc25a43	Slc2a4
Slc14a1	Slc1a4	Slc24a5	Slc25a44	Slc2a4rg-ps
Slc14a2	Slc1a5	Slc24a6	Slc25a45	Slc2a5
Slc15a1	Slc1a6	Slc25a1	Slc25a46	Slc2a6
Slc15a2	Slc1a7	Slc25a10	Slc25a47	Slc2a7
Slc15a3	Slc20a1	Slc25a11	Slc25a48	Slc2a8
Slc15a4	Slc20a2	Slc25a12	Slc25a5	Slc2a9
Slc15a5	Slc22a1	Slc25a13	Slc25a51	Slc30a1
Slc16a1	Slc22a12	Slc25a14	Slc25a53	Slc30a10
Slc16a10	Slc22a13	Slc25a15	Slc25a54	Slc30a2
Slc16a11	Slc22a13b-ps	Slc25a16	Slc26a1	Slc30a3
Slc16a12	Slc22a14	Slc25a17	Slc26a10	Slc30a4
Slc16a13	Slc22a15	Slc25a18	Slc26a11	Slc30a5
Slc16a14	Slc22a16	Slc25a19	Slc26a2	Slc30a6
Slc16a2	Slc22a17	Slc25a2	Slc26a3	Slc30a7
Slc16a3	Slc22a18	Slc25a20	Slc26a4	Slc30a8
Slc16a4	Slc22a19	Slc25a21	Slc26a5	Slc30a9

Slc31a1	Slc37a2	Slc44a3	Slc5a7	Slc7a9
Slc31a2	Slc37a3	Slc44a4	Slc5a8	Slc8a1
Slc32a1	Slc37a4	Slc44a5	Slc5a9	Slc8a2
Slc33a1	Slc38a1	Slc45a1	Slc6a1	Slc8a3
Slc34a1	Slc38a10	Slc45a2	Slc6a11	Slc8b1
Slc34a2	Slc38a11	Slc45a3	Slc6a12	Slc9a1
Slc34a3	Slc38a2	Slc45a4	Slc6a13	Slc9a2
Slc35a1	Slc38a3	Slc46a1	Slc6a14	Slc9a3
Slc35a2	Slc38a4	Slc46a2	Slc6a15	Slc9a3r1
Slc35a3	Slc38a5	Slc46a3	Slc6a16	Slc9a3r2
Slc35a4	Slc38a6	Slc47a1	Slc6a17	Slc9a4
Slc35a5	Slc38a7	Slc47a2	Slc6a18	Slc9a5
Slc35b1	Slc38a8	Slc48a1	Slc6a19	Slc9a6
Slc35b2	Slc38a9	Slc49a4	Slc6a19os	Slc9a7
Slc35b3	Slc39a1	Slc4a1	Slc6a2	Slc9a8
Slc35b4	Slc39a1-ps	Slc4a10	Slc6a20a	Slc9a9
Slc35c1	Slc39a10	Slc4a11	Slc6a20b	Slc9b1
Slc35c2	Slc39a11	Slc4a1ap	Slc6a21	Slc9b2
Slc35d1	Slc39a12	Slc4a2	Slc6a3	Slc9c1
Slc35d2	Slc39a13	Slc4a3	Slc6a4	Slco1a1
Slc35d3	Slc39a14	Slc4a4	Slc6a5	Slco1a4
Slc35e1	Slc39a2	Slc4a5	Slc6a6	Slco1a5
Slc35e2	Slc39a3	Slc4a7	Slc6a7	Slco1a6
Slc35e3	Slc39a4	Slc4a8	Slc6a8	Slco1b2
Slc35e4	Slc39a5	Slc4a9	Slc6a9	Slco1c1
Slc35f1	Slc39a6	Slc50a1	Slc7a1	Slco2a1
Slc35f2	Slc39a7	Slc51a	Slc7a10	Slco2b1
Slc35f3	Slc39a8	Slc51b	Slc7a11	Slco3a1
Slc35f4	Slc39a9	Slc52a2	Slc7a12	Slco4a1
Slc35f5	Slc3a1	Slc52a3	Slc7a13	Slco4c1
Slc35f6	Slc3a2	Slc5a1	Slc7a14	Slco5a1
Slc35g1	Slc40a1	Slc5a10	Slc7a15	Slco6b1
Slc35g2	Slc41a1	Slc5a11	Slc7a2	Slco6c1
Slc35g3	Slc41a2	Slc5a12	Slc7a3	Slco6d1
Slc36a1	Slc41a3	Slc5a2	Slc7a4	
Slc36a1os	Slc43a1	Slc5a3	Slc7a5	
Slc36a2	Slc43a2	Slc5a4a	Slc7a6	
Slc36a3	Slc43a3	Slc5a4b	Slc7a6os	
Slc36a4	Slc44a1	Slc5a5	Slc7a7	
Slc37a1	Slc44a2	Slc5a6	Slc7a8	

Supplementary Data 2 **HIF1-driven genes in T cells** – genes sig ↑ in PHD KO vs PHD WT T cells AND sig ↓ in HIF1 KO vs HIF WT T cells (adjusted $p<0.05$)

Gene	**WT vs PHD KO** (GSE85131)		**WT vs HIF 1 KO** (GSE29765)	
	**log2 FC**	**adjusted *p***	**log2 FC**	**adjusted *p***
**Adm**	-2.546121	0.007924888	3.12194	0.0102
**Ak4**	-1.889304	0.03409644	5.425637	1.45E-08
**Aldoa**	-1.120577	0.001869517	1.726133	1.03E-05
**Ankrd37**	-1.569994	0.03531962	5.4514	2.44E-07
**Anxa2**	-2.282505	0.002006899	1.92107	1.22E-05
**Anxa4**	-2.676462	0.000389229	0.7439367	0.00254
**Arhgdig**	-2.939961	0.01362047	0.9907433	0.000249
**Arl6ip5**	-0.5176526	0.04086054	0.3694333	0.0197
**Atg9b**	-1.305052	0.03531962	0.9797667	0.000519
**Basp1**	-2.522154	0.04120269	0.9180667	0.0025
**Bend5**	-2.498746	0.01618295	3.501797	1.21E-06
**Bhlhe40**	-2.411854	0.03742782	1.365967	2.99E-05
**Bnip3**	-3.053233	0.000634716	5.448083	2.44E-07
**Bnip3l**	-1.628323	0.01016226	1.60777	3.24E-05
**Bsg**	-1.679345	0.001869517	1.578307	9.69E-06
**Casp6**	-2.026736	0.008188236	0.5074533	0.00545
**Ccdc115**	-0.986874	0.02334512	0.84104	0.0025
**Cd82**	-0.9546427	0.02270512	0.3532333	0.0206
**Cdkn1a**	-2.602944	0.008798899	1.527493	0.00168
**Cflar**	-1.028831	0.03903261	0.6648467	0.0142
**Clcn3**	-1.371524	0.01656015	1.796747	1.42E-05
**Clybl**	-1.641156	0.01435681	2.821843	1.55E-06
**Cnot7**	-0.5462343	0.03253053	0.4298833	0.0337
**Cysltr2**	-3.098109	0.003217063	0.5772233	0.0304
**Dnajc21**	-0.8489705	0.01895011	0.4185967	0.0117
**Egln1**	-1.321645	0.01321657	2.560813	2.49E-06
**Egln3**	-2.864769	0.004336541	2.348133	0.000108
**Eif4ebp1**	-1.474452	0.02403916	0.58393	0.0252
**Ero1l**	-2.322111	0.00221171	2.095583	7.55E-05
**Espn**	-1.122276	0.01652142	1.378443	0.0137
**Exoc2**	-1.114776	0.03903261	0.4438	0.0462
**Fam162a**	-1.76144	0.01866003	1.626167	4.81E-06
**Fam57a**	-2.34598	0.04001082	0.8791733	0.00179
**Fscn1**	-1.418827	0.01070897	1.9271	0.00221
**Gapdh**	-1.267996	0.006960698	0.6893	0.000838
**Gata3**	-2.158387	0.002293318	0.6264567	0.0177
**Gpi1**	-0.9910033	0.02270512	1.62034	6.17E-06

**Gtf2e2**	-1.06866	0.0288444	0.7408933	0.00515
**Hes6**	-0.6589254	0.03992262	1.002093	0.000859
**Hfe**	-1.77452	0.01941582	1.38228	0.00172
**Higd1a**	-2.001226	0.03680992	1.1232	6.33E-05
**Hilpda**	-3.157958	0.008762919	2.75782	2.42E-05
**Hk2**	-2.108636	0.02352318	1.86932	0.000261
**Id2**	-1.795515	0.03798084	0.3579667	0.0359
**Ier3**	-3.735563	0.006065698	1.900093	9.69E-06
**Il10ra**	-1.680866	0.04986422	0.9049667	0.00226
**Itga7**	-2.825229	0.04122895	4.36966	4.51E-06
**Jmjd6**	-1.418683	0.005851341	2.029273	1.74E-06
**Kcnk7**	-2.131209	0.02248697	1.429847	6.27E-05
**Kctd14**	-1.085374	0.02333753	0.3458133	0.0318
**Klk8**	-1.126952	0.02890286	0.9410067	0.000807
**Ldha**	-0.971314	0.008058179	0.5876333	0.00172
**Lgals3**	-2.446662	0.001790329	0.5862333	0.00527
**Lgals7**	-2.146141	0.00999231	1.284053	2.55E-05
**Map2k1**	-0.9446497	0.009473778	1.22525	0.000221
**Mboat2**	-2.913038	0.01648667	3.057593	1.84E-06
**Mgarp**	-4.287145	0.00113808	5.92323	5.94E-07
**Mt1**	-6.238007	0.00106782	3.816203	1.97E-06
**Mxi1**	-1.981789	0.02860696	0.88402	0.00117
**Ndrg1**	-1.542519	0.02011137	2.41606	0.000284
**Nfil3**	-2.494121	0.002943253	0.98991	0.00192
**Npc2**	-0.684478	0.0288444	0.5924367	0.01
**P4ha2**	-4.49362	0.003413915	4.705813	7.67E-07
**Pafah1b3**	-2.07933	0.002942767	1.27817	2.56E-05
**Pdxp**	-1.409388	0.04376749	2.008313	6.48E-06
**Pgk1**	-1.411721	0.000348914	0.9841333	0.000134
**Pgm2**	-1.616367	0.01941582	2.98594	9.97E-07
**Pla2g12a**	-1.613231	0.0167328	0.6175733	0.00699
**Plekha2**	-1.157862	0.01586774	0.5278367	0.0154
**Plod2**	-2.005124	0.02035961	2.016443	2.56E-05
**Plod3**	-1.13711	0.008393207	0.5608333	0.00653
**Ppp1r3b**	-1.419394	0.04986422	2.6209	0.000106
**Prdx5**	-0.6216044	0.04399256	0.47666	0.0131
**Prelid1**	-0.6593593	0.002942767	0.8742	0.000185
**Prelid2**	-2.23291	0.009560788	2.200853	1.55E-06
**Prkcdbp**	-3.930553	0.002571161	1.334133	0.00181
**Pygl**	-2.393317	0.01147757	3.225617	5.23E-05
**R3hdm1**	-0.7241527	0.03798329	0.8591667	0.00138
**Rab33a**	-3.272228	0.003413915	3.181063	4.49E-05

**Rcor2**	-2.149705	0.01312204	0.8362167	0.0186
**Rnf126**	-1.152552	0.01106983	1.041323	0.000194
**Rnf208**	-1.837556	0.02164251	0.5452167	0.00641
**Sap30**	-1.66957	0.004884304	1.023367	0.000918
**Sdc4**	-2.477841	0.04334791	3.80822	6.80E-06
**Selenbp1**	-3.411241	0.003062118	5.339177	2.49E-06
**Selp**	-3.176499	0.005393006	0.8426067	0.00769
**Slc16a3**	-2.54334	0.005213429	3.239053	1.40E-05
**Slc2a3**	-3.904958	0.03024235	4.698387	6.67E-07
**Slc6a6**	-0.6341538	0.004714412	0.3532667	0.0357
**Smtnl2**	-3.354	0.00957543	3.015877	3.24E-05
**Spsb1**	-2.956118	0.01987694	0.3806633	0.0331
**St3gal1**	-1.233001	0.02620593	0.36014	0.0196
**Stat5a**	-1.22865	0.04244288	0.8156233	0.00185
**Stc2**	-2.925523	0.008188236	0.38238	0.0306
**Syce2**	-1.45646	0.009473778	1.17723	3.06E-05
**Tex264**	-0.5523263	0.04738994	0.46934	0.00701
**Tgm2**	-3.012663	0.01941582	0.81274	0.00579
**Tmem115**	-0.6638337	0.02391275	0.32676	0.031
**Tmem45a**	-1.966615	0.006960698	0.8382767	0.0162
**Tmem74b**	-2.039036	0.03085073	0.4033033	0.0468
**Tpi1**	-1.38717	0.005393006	2.208067	1.02E-06
**Tubb6**	-1.359832	0.03742782	0.9221367	0.000221
**Upp1**	-3.950855	0.005393006	0.55717	0.0167
**Vdac1**	-0.9655575	0.001790329	0.7574333	0.00141
**Vegfa**	-2.144259	0.03903261	2.48201	5.23E-05
**Vhl**	-1.206885	0.014941	0.95558	0.00227
**Vldlr**	-2.827102	0.005458818	4.121637	7.85E-07
**Xpnpep2**	-1.915553	0.03516309	0.3893867	0.0207

Supplementary Data 3 **HIF1 metabolic gene signature** (adjusted *p*<0.05)

Gene	**WT vs PHD KO** (GSE85131)		**WT vs HIF 1 KO** (GSE29765)	
	**log2 FC**	**adjusted *p***	**log2 FC**	**adjusted *p***
**Ak4**	-1.889304	0.03409644	5.425637	1.45E-08
**Aldoa**	-1.120577	0.00186952	1.726133	1.03E-05
**Gapdh**	-1.267996	0.0069607	0.6893	0.000838
**Gpi1**	-0.991003	0.02270512	1.62034	6.17E-06
**Hk2**	-2.108636	0.02352318	1.86932	0.000261
**Ldha**	-0.971314	0.00805818	0.5876333	0.00172
**Mboat2**	-2.913038	0.01648667	3.057593	1.84E-06
**P4ha2**	-4.49362	0.00341392	4.705813	7.67E-07
**Pafah1b3**	-2.07933	0.00294277	1.27817	2.56E-05
**Pdxp**	-1.409388	0.04376749	2.008313	6.48E-06
**Pgk1**	-1.411721	0.00034891	0.9841333	0.000134
**Pgm2**	-1.616367	0.01941582	2.98594	9.97E-07
**Pla2g12a**	-1.613231	0.0167328	0.6175733	0.00699
**Plod2**	-2.005124	0.02035961	2.016443	2.56E-05
**Plod3**	-1.13711	0.00839321	0.5608333	0.00653
**Pygl**	-2.393317	0.01147757	3.225617	5.23E-05
**Slc16a3**	-2.54334	0.00521343	3.239053	1.40E-05
**Slc2a3**	-3.904958	0.03024235	4.698387	6.67E-07
**Slc6a6**	-0.634153	0.00471441	0.3532667	0.0357
**St3gal1**	-1.233001	0.02620593	0.36014	0.0196
**Tpi1**	-1.38717	0.00539301	2.208067	1.02E-06
**Upp1**	-3.950855	0.00539301	0.55717	0.0167

<u>Supplementary Data 4</u> Peptide sequence for vectors

1. Peptide sequence for vector encoding mouse PDXP

MNPAISVALLLSVLQVSRGQKVTSLTACLVNQNLRLDCRHENNTKDNSIQHEFSLTREKR
KHVLSGTLGIPEHTYRSRVTLSNQPYIKVLTLANFTTKDEGDYFCELRVSGANPMSSNKSIS
VYRDKLVKCGGISLLVQNTSWMLLLLLSLSLLQALDFISLRAKRGSGATNFSLLKQAGDV
EENPGPMARCERLRGAALRDVLGQAQGVLFDCDGVLWNGERIVPGAPELLQRLARAGK
NTLFVSNNSRRARPELALRFARLGFAGLRAEQLFSSALCAARLLRQRLSGPPDASGAVFVL
GGEGLRAELRAAGLRLAGDPGEDPRVRAVLVGYDEQFSFSRLTEACAHLRDPDCLLVAT
DRDPWHPLSDGSRTPGTGSLAKKVETASGRQALVVGKPSPYMFQCITEDFSVDPARTLMV
GDRLETDILFGHRCGMTTVLTLTGVSSLEEAQAYLTAGQRDLVPHYYVESIADLMEGLED

**Protein Name**	**NCBI Reference Sequence**
Thy-1.1	**AAR17087.1**
Furin cleavage site	RAK**R** (cleavage after **R**)
Picornavirus 2A self-cleaving sequence	GSGATNFSLLKQAGDVEENP**GP**<br>(cleavage occurs between **G** and **P**)
PDXP	NP_064667.2
KK Alanine residues substituted to lysine residues	Decreased affinity to pyridoxal phosphate(*Kestler et al. 2014*)

2. Peptide sequence for vector encoding mouse PDXK

MNPAISVALLLSVLQVSRGQKVTSLTACLVNQNLRLDCRHENNTKDNSIQHEFSL
TREKRKHVLSGTLGIPEHTYRSRVTLSNQPYIKVLTLANFTTKDEGDYFCELRVSG
ANPMSSNKSISVYRDKLVKCGGISLLVQNTSWMLLLLLSLSLLQALDFISLRAKRG
SGATNFSLLKQAGDVEENPGPMEGECRVLSIQSHVVRGYVGNRAAMFPLQVLGFEVDAVNS
VQFSNHTGYAHWKGQVLKSQELHELYEGLKVNDVNKYDYVLTGYTRDKSFLAMVVDIVRELKQQ
NSRLVYVCDPVMGDKWNGEGSMYVPQDLLPVYRDKVVPVADIITPNQFEAELLSGRKIHSQEEAFE
VMDMLHCMGPDTVVITSSDLPSSQGSDYLIALGSQRMRKPDGSTVTQRIRMEMRKVETVFVGTGD
LFAAMLLAWTHKHPDNLKVACEKTVSAMQHVLQRTIRCAKAEAGEGQKPSPAQLELRMVQSKRD
IEDPEIVVQATVL

**Protein Name**	**NCBI Reference Sequence**
Thy-1.1	**AAR17087.1**
Furin cleavage site	RAK**R** (cleavage after **R**)
Picornavirus 2A self-cleaving sequence	GSGATNFSLLKQAGDVEENP**GP**<br>(cleavage occurs between **G** and **P**)
PDXK	NP_742146.1
T Alanine residues substituted to threonine	decreased pyridoxal kinase activity (*Chelban et al. 2019*)
