# Supplementary figures and images for: Vitamin B6 Metabolism Determines T Cell Anti-Tumor Responses
Source: Front Immunol. 2022 Feb 17;13:837669. doi: 10.3389/fimmu.2022.837669 (PMC8891565; doi:10.3389/fimmu.2022.837669)

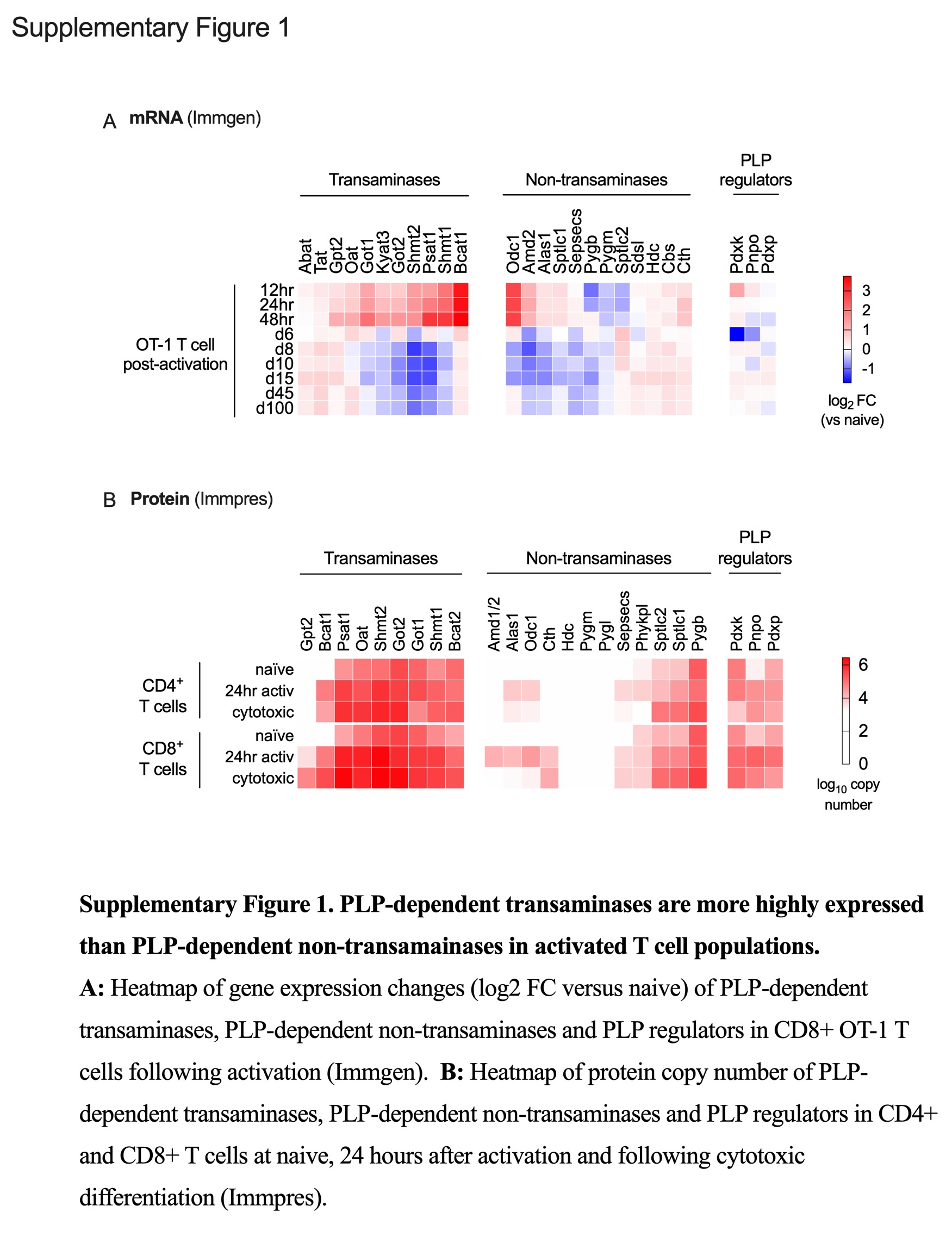

Supplement: Supplementary file 2 [file Image_1.jpeg]

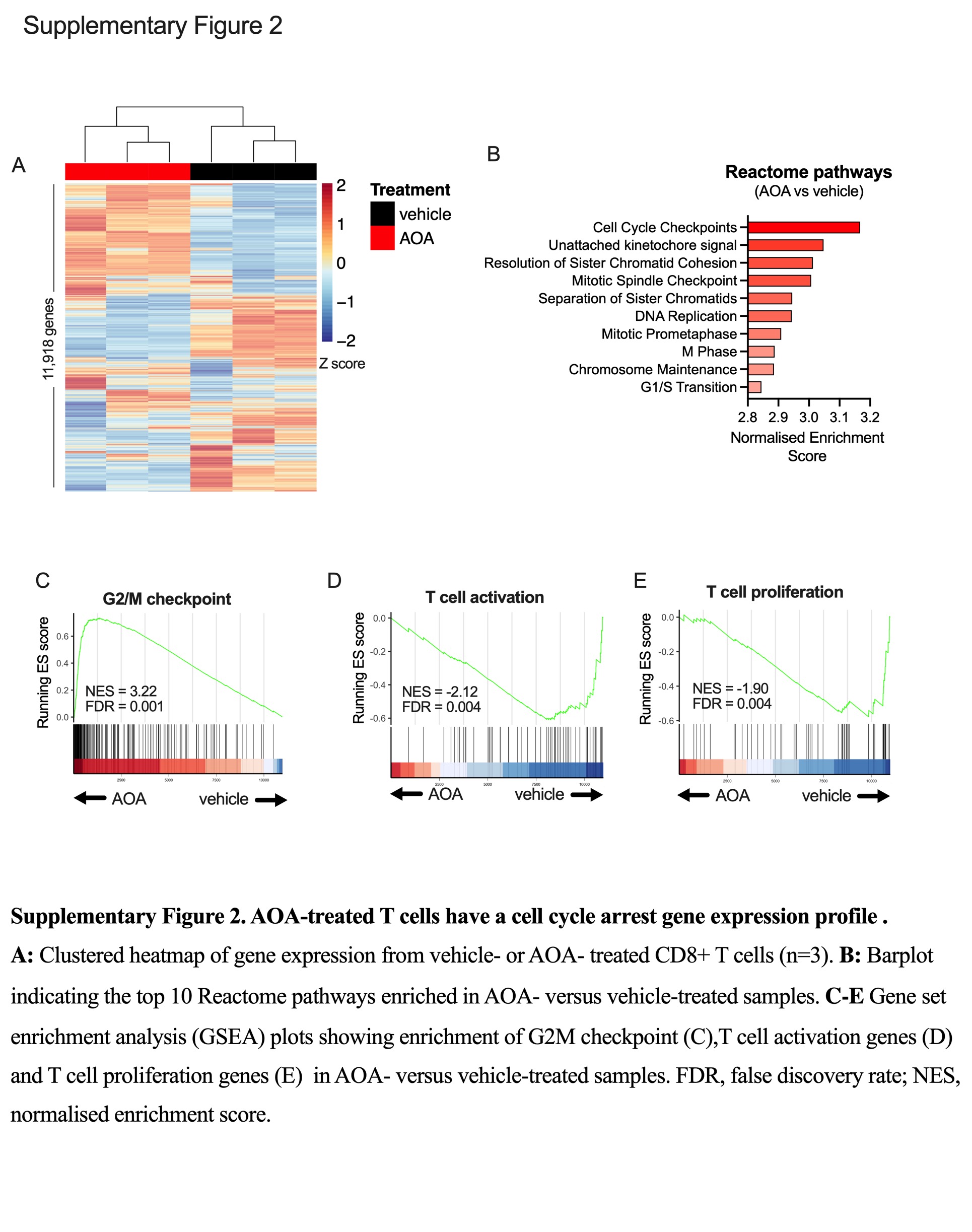

Supplement: Supplementary file 3 [file Image_2.jpeg]

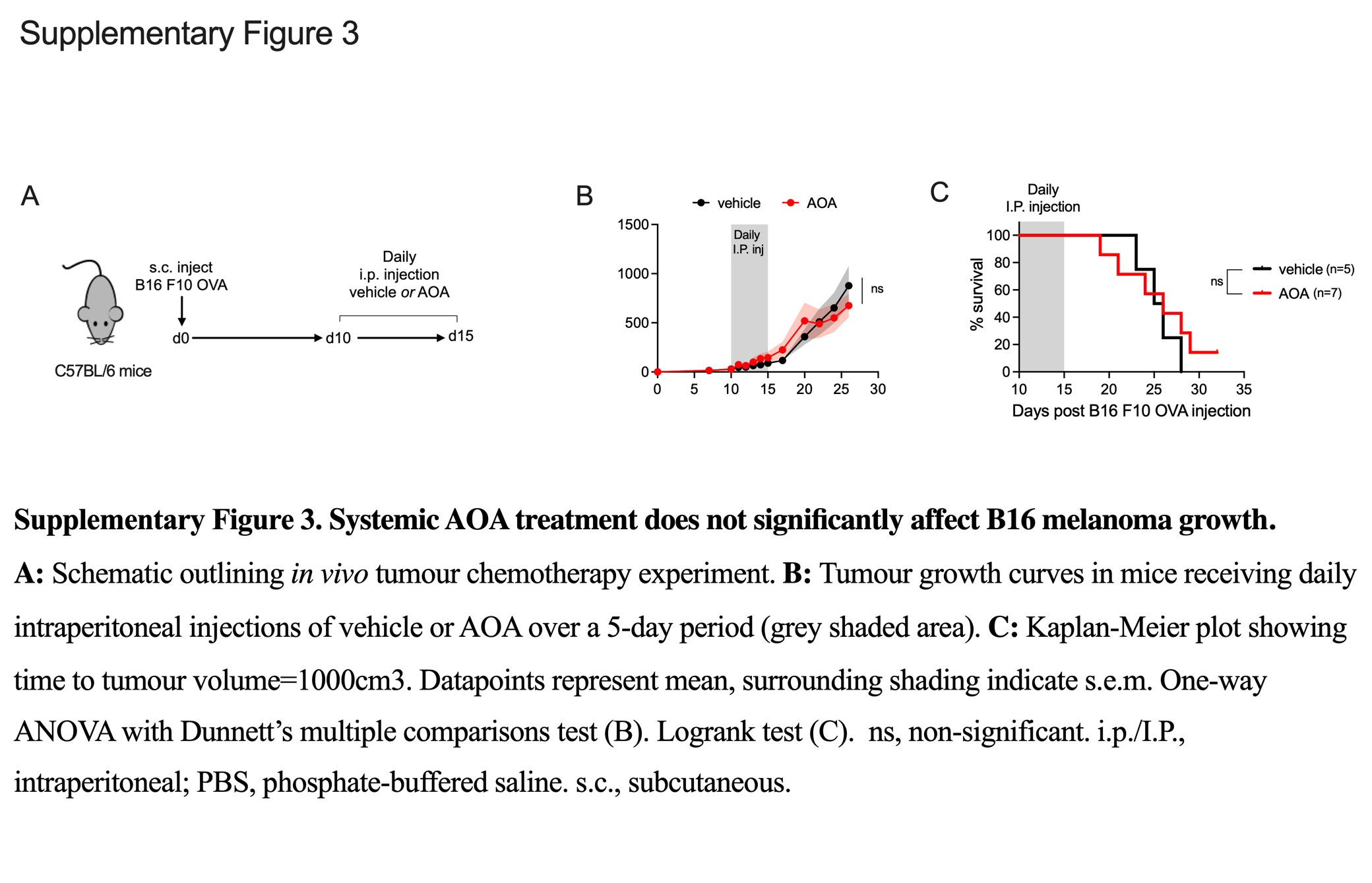

Supplement: Supplementary file 4 [file Image_3.jpeg]

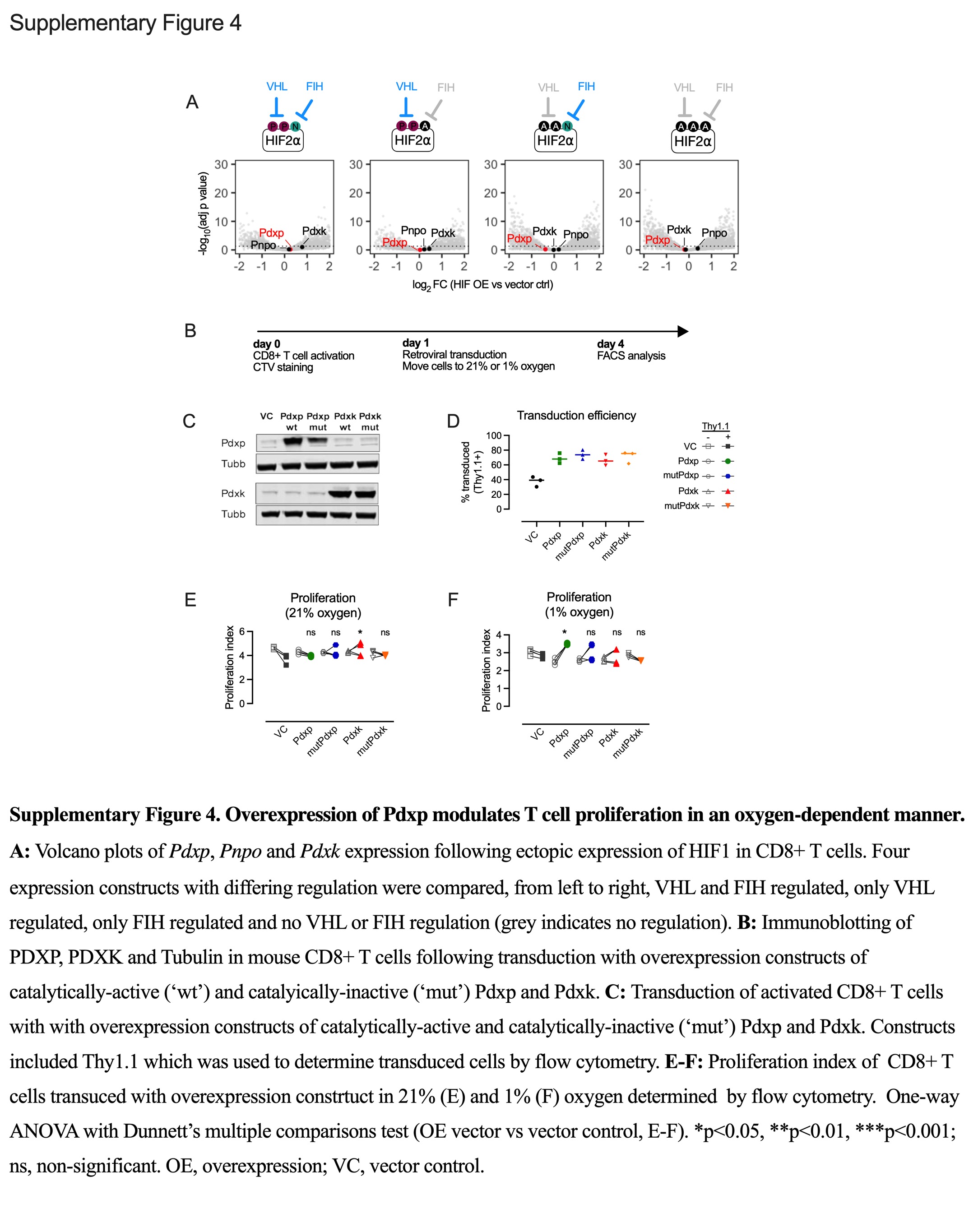

Supplement: Supplementary file 5 [file Image_4.jpeg]
